# Deep Learning-Based Obesity Identification System for Young Adults Using Smartphone Inertial Measurements

**DOI:** 10.3390/ijerph21091178

**Published:** 2024-09-04

**Authors:** Gou-Sung Degbey, Eunmin Hwang, Jinyoung Park, Sungchul Lee

**Affiliations:** 1 Division of Computer Science and Engineering, Sunmoon University, Asan 31460, Republic of Korea; gousungdegbey17@sunmoon.ac.kr; 2 William F. Harrah College of Hotel Administration, University of Nevada Las Vegas, Las Vegas, NV 89154, USA; eunmin.hwang@unlv.edu; 3 College of Nursing, Yonsei University, 50 Yonsei-ro, Seodaemun-gu, Seoul 03722, Republic of Korea; jyp0766@gmail.com

**Keywords:** obesity recognition, gait analysis, deep learning, mobile health applications, AI

## Abstract

Obesity recognition in adolescents is a growing concern. This study presents a deep learning-based obesity identification framework that integrates smartphone inertial measurements with deep learning models to address this issue. Utilizing data from accelerometers, gyroscopes, and rotation vectors collected via a mobile health application, we analyzed gait patterns for obesity indicators. Our framework employs three deep learning models: convolutional neural networks (CNNs), long-short-term memory network (LSTM), and a hybrid CNN–LSTM model. Trained on data from 138 subjects, including both normal and obese individuals, and tested on an additional 35 subjects, the hybrid model achieved the highest accuracy of 97%, followed by the LSTM model at 96.31% and the CNN model at 95.81%. Despite the promising outcomes, the study has limitations, such as a small sample and the exclusion of individuals with distorted gait. In future work, we aim to develop more generalized models that accommodate a broader range of gait patterns, including those with medical conditions.

## 1. Introduction

According to the World Health Organization (WHO) [1], body mass index (BMI) serves as a straightforward metric for weight-for-height assessment and is widely employed for categorizing overweight and obesity in adolescents and adults. It is quantified as an individual’s weight in kilograms divided by the square of their height in meters (kg/m^2^). As age increases, the health risks associated with high BMI also increase [2]. A 2022 WHO report [3] stated that one in eight people worldwide are living with obesity. Since 1990, global adult obesity has more than doubled and adolescent obesity has quadrupled. In the same year, 2.5 billion adults (18 years and older) were overweight, with 890 million of them living with obesity. Additionally, 37 million children under 5 years were overweight, and over 390 million children and adolescents aged 5–19 years were overweight, including 160 million living with obesity. A report from the Telegraph newspaper [4] highlighted that obesity now poses a greater threat to global health than hunger, as per a new Lancet study. Given the rapid rise in overweight and obesity, not only in adults but more importantly in younger populations, it is crucial to explore possible ways for effective early identification and intervention strategies. While traditional methods of obesity identification such as the BMI provide a straightforward metric for categorizing individuals based on weight and height, it is a relatively crude measure that fails to capture the nuanced biomechanical changes associated with obesity, particularly those reflected in gait patterns. Obesity is linked with numerous health conditions, including osteoarthritis [5], sleep apnea [6], cancer [7], and mental illnesses such as depression [8] and anxiety [9].

Gait is an essential function enabling movement within the environment and between locations. It results from the coordinated movements of body segments, driven by the interaction of internal and external factors, and is regulated by the neuromusculoskeletal system. Normal gait is characterized by its stability and flexibility, allowing for adjustments in speed and navigation across various terrains while maintaining energy efficiency. Obesity has been found to influence an individual’s manner of walking, including joint and walking velocities [10,11], ankle joint speed [12], peak extensor knee movements [13], and knee joint loads [14]. Human balance is maintained by a complex set of sensorimotor and musculoskeletal systems controlling vision, proprioception, vestibular function, muscle contraction, and more. Balance is used to diagnose disorders and diseases related to the nervous system [15], such as ataxia [16], cognitive deficits [17,18], Parkinson’s disease [19], and vision problems [20]. Gait information can be captured using several sensing modalities such as wearable sensors attached to the body, including accelerometers, gyroscopes, and pressure sensors. While these sensors are effective, they may be inconvenient for daily use and are often reliant on laboratory-based analysis systems, which, while accurate, are often expensive, time-consuming, and impractical for large-scale or real-time monitoring. This study seeks to address these limitations by exploring the use of smartphone inertial sensors combined with deep learning models to identify obesity through gait analysis. Unlike BMI, which offers a static measure, gait analysis provides more dynamic insights into how obesity affects movements, potentially allowing for earlier detection. This method could help raise awareness of weight changes and aid in obesity prevention by providing quick alerts, enabling individuals to monitor their health more effectively. This study proposes a novel framework for obesity identification in adolescents using gait data collected from smartphone inertial sensors. The primary objective of this study is to investigate whether gait patterns, captured smartphone inertial sensors, can be used to accurately identify differences in gait patterns between a normal group and an obese group. Specifically, this study evaluates the performance of three deep learning models: convolutional neural networks (CNNs), long short-term memory (LSTM), and a hybrid CNN–LSTM model. These models are chosen for their ability to capture both spatial and temporal patterns in sequential data, making them well suited for analyzing the complex gait patterns associated with obesity. While deep learning models have been widely used in various domains, their application in obesity identification using gait data remains underexplored. This study contributes to the field by demonstrating how these models can be effectively applied to real-world, smartphone-collected data, providing a more accessible and scalable approach to obesity monitoring. By integrating deep learning with smartphone technology, this research offers a practical solution that can be deployed in everyday settings, potentially enabling continuous and non-intrusive monitoring of obesity risk in adolescents.

## 2. Related Work and Literature Survey

### 2.1. Gait Analysis Techniques and Limitations

Human gait, a periodic motion of body segments, is analyzed through a process called gait analysis. This technique has gained significant popularity due to its applications in clinical diagnosis, robotics, sports, and biomechanics. It encompasses various methods for studying human walking patterns and biomechanics.

One of the most notable techniques is motion capture [21], which use markers placed on the body to track the movement of specific body segments during walking, providing highly accurate data by capturing joint angles and movements in three dimensions. However, this method is expensive and requires a specialized laboratory environment. Force plates [22] measure ground reaction forces and moments during walking, offering valuable insights about balance, stability, and propulsion. Despite their benefits, force plates are also costly and require careful setup and calibration. Electromyography (EMG) [23] measures electrical activity in muscles during walking, providing information on muscle activation patterns and timing. While it allows assessment of muscle recruitment, coordination, and timing, it can be uncomfortable or intrusive due to the placement of electrodes on muscles and is prone to signal contamination from nearby muscles. Wearable sensors, such as accelerometers [24], gyroscopes [25], and inertial measurement units (IMUs) [26], are attached to the body to measure movement and orientation during walking. This technique is portable and unobtrusive, allowing gait analysis in real-world settings, capturing spatiotemporal parameters like step length, cadence, and gait agility. However, data quality can be affected by sensor placement.

While these methods have significantly advanced our understanding of human gait, they are not without limitations. The reliance on laboratory-based systems like motion capture and force plates restricts the scalability of gait analysis, making it challenging for widespread public health applications. Wearable sensors, despite their advantages, are mainly used in clinical diagnosis, leaving a gap in research exploring their utility for continuous, real-world obesity monitoring. This study aims to bridge this gap by leveraging smartphone-based inertial sensors, combining the portability of wearable devices with the accessibility of everyday technology. This approach addresses the limitations of previous studies by offering a more practical and widely applicable method for gait analysis, particularly in the context of obesity detection.

### 2.2. Smartphone-Based Gait Analysis for Obesity Recognition

With the increasing number of studies involving gait analysis, some researchers have applied it to obesity recognition, yielding promising results. Subhrangshu et al. [26] developed a smartphone-based IoT framework attached to participants’ chests. Using machine learning and deep learning models, it monitored the gait of subjects and predicted their BMI. Data from 30 different individuals were collected using smartphone inertial sensors, such as the accelerometer and gyroscope. They employed an edge computing technique using incremental machine learning coupled with the stochastic gradient descent algorithm, achieving 98.6% accuracy. This study demonstrates the potential of smartphone-based gait analysis for obesity detection, but is limited primarily to the small sample.

Murtha et al. [27] developed a framework for predicting weight gain in young adults with overweight and obesity using electronic health records (EHRs) and machine learning models. Analyzing data from 24,183 participants aged 18 to 39, they found that age, sex, and baseline BMI were the most important variables. However, the machine learning models performed poorly, highlighting the limitations of using EHR data alone for obesity recognition. This suggests that while EHRs provide valuable longitudinal data, they may need to be complemented with more dynamic measures such as gait analysis for better obesity prediction.

Steinberg et al. [28] and Rosso et al. [29] conducted studies exploring the relationship between BMI and gait characteristics. Steinberg et al. found that overweight individuals exhibited greater hip joint angles and shorter knee and ankle trajectories, while Rosso et al. identified specific joint kinematic parameters associated with BMI. These studies reinforce the link between BMI and altered gait patterns, but primarily focus on controlled environments, limiting their applicability to real-world settings. Lee et al. [30,31] investigated the relationship between obesity and walking patterns. They developed an mHealth application to collect gait data from 244 high school students. Using rotation vector sensor data and applying feedforward deep neural networks and deep convolutional neural networks, the models distinguished walking patterns between non-obese and obese groups with 90.5% accuracy, indicating a relationship between walking patterns and obesity.

Building on these findings, our study expands on the work of Lee et al. [31] by using smartphone-based gait analysis to explore relationships between gait characteristics and BMI. Gait data were collected from 244 first-year high school students using an mHealth application installed on a smartphone, and a sample of 173 students. By leveraging smartphone-based IMUs, along with a hybrid deep learning model, we achieved a higher accuracy of 97%. The remainder of this paper is organized as follows. Section 3 introduces the dataset acquisition method and deep learning techniques used, Section 4 reports the experiments and results, Section 5 provides a discussion of the findings, and Section 6 concludes with suggestions for future work.

## 3. Methodology

### 3.1. Data Collection and Participants Demographic

**Figure 1:**
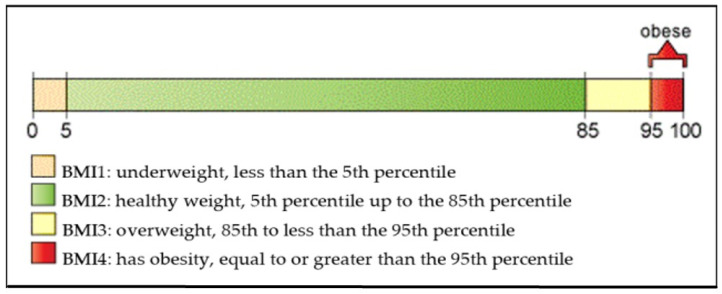
CDC BMI percentile calculator for children and teens (Courtesy of [31]).

In this study, we calculated the body mass index (BMI) for each participant using the BMI calculator provided by the Centers for Disease Control and Prevention (CDC) [32], following a similar approach to Lee et al. [31]. Participants self-reported their height and weight for BMI calculations. As shown in Figure 1,BMI was categorized into four groups based on the CDC’s BMI-for-age growth charts: BMI 1 represents the underweight category with BMI percentage below the 5th percentile (BMI% < 5th percentile); BMI 2 represents the normal-weight category with BMI percentage between the 5th and the 85th percentile (5th percentile < BMI% < 85th percentile); BMI 3 represents the overweight category with BMI percentage between the 85th percentile and the 95th percentile (85th percentile < BMI% < 95th percentile) and BMI 4 represents the obese category with BMI percentage above the 95th percentile (BMI% > 95th percentile).

To capture and analyze gait patterns, a mobile health application was developed by Lee et al. [30]. This application was originally designed to measure and detect differences in walking movements between patients with traumatic brain injuries and healthy individuals. The application leverages smartphone inertial sensors to collect gait data, making it highly suitable for real-time gait analysis. The smartphone-based inertial motion sensors used included an accelerometer, gyroscope, and rotation vector. These sensors are commonly used together in gait analysis, motion tracking, and activity recognition, providing a comprehensive understanding of gait and body movements. The rotation vector [33] allows basic analysis by calculating the variation in X, Y, and Z rotations, which shows walking movements over time. A total of 244 first-year high school students were initially enrolled in the data collection phase. However, 71 students were excluded from this study due to preexisting medical conditions that could distort gait patterns, resulting in a final sample of 173 participants. During the data collection process, all participants wore the smartphone in a waistband pocket positioned at the center of the body, with the smartphone device placed horizontally and facing forward. The participants walked in a straight line along a 78-foot course, turned around, and returned to the starting point. The smartphone collected data at a sampling rate of 100 Hz, providing the foundation for analysis using deep learning models described in the following sections. The demographic information of the students is presented in Table 1.

### 3.2. Data Preprocessing

The data collected around the rotation vector sensor, especially the rotation matrix, are the main data used for the purpose of this study. In the preprocessing step, we applied denoising to the data, as smartphone inertial sensors are commonly prone to external noise. To address this, we used a moving average filter algorithm [34]. The moving average filter is a common technique used in signal processing to smooth out fluctuations or noise in a dataset, particularly in time series data. It works by calculating the average value of a series of data points within a specified window size and replaces the original value with this calculated average. The moving average filter process is described by the formula below. This process is performed iteratively across the entire dataset, creating a smoothed version of the original signal. In our case, we applied the moving average with a rolling window of size 15, meaning that at each iteration, the average of the 15 points are computed and this process is repeated across the entire dataset.

(1) $$\begin{array}{c} SMA_{k\;}=\frac{p_{n-k+1}+p_{n-k+2}+\ldots +p_{n}\;}{k}\\ =\frac{1}{k}\;\sum_{i=n-k+1}^{n}\;pi \end{array}$$

### 3.3. Data Normalization

The data were then normalized using the MinMaxScaler algorithm from the sklearn library. The MinMaxScaler [35] algorithm is a normalization technique, widely employed in data preprocessing, offering a systematic approach to scale numeric features within a predetermined range. The method operates by rescaling the features of the dataset to fit within a specified interval, typically between 0 and 1. The operational mechanism of MinMaxScaler hinges upon a simple transformation process. It computes the minimum and the maximum values of each feature within the dataset, and subsequently applies a linear transformation to map feature values to the designated range. Mathematically, this transformation is represented as follows:

(2) $$x_{scaled}=\frac{x-x_{min}}{x_{max}-x_{min}}$$

### 3.4. Data Segmentation

**Figure 2:**
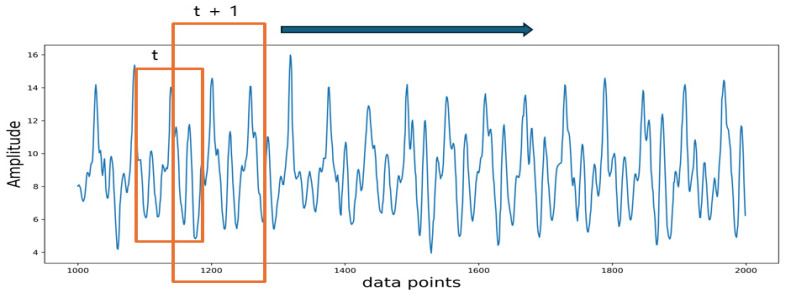
Data segmentation process. The orange frame highlights the overlapping area used in data segmentation, with the arrow indicating that this overlap is applied consistently throughout the dataset.

The input to the model consists of sequence data, which is a short time series extracted from the raw sensor data. During data collection, the data were recorded continuously. In order to preserve the temporal relationship between the data points collected while providing sufficient data points for model training, time series segmentation was applied to the continuous data. A sliding window method was employed to segment the entire data into sequential segments. A fixed window size of 100 data points is used with an overlap of 50%. The segmentation process is shown in Figure 2.

### 3.5. Deep Learning Models

Deep learning has played a significant role in classification and recognition in various time series-related tasks. In this study, deep learning models were implemented to classify data between normal and overweight/obese groups. The models were deep convolutional neural networks (DCNNs), long short-term memory (LSTM), and a hybrid combination of deep convolutional neural networks and long short-term memory.

#### 3.5.1. Deep Convolutional Neural Networks

Deep convolutional neural networks (DCNNs) have become fundamental in deep learning, revolutionizing various domains of artificial intelligence. Prominent applications of CNNs include image classification and segmentation, object detection, video processing, natural language processing, and speech recognition. The powerful capability of DCNNs is mainly attributed to multiple feature extraction stages that can automatically learn representations from data. However, although CNNs can learn complex objects or patterns in images or videos, they may not be suitable for numerous applications involving 1D signals. To address this issue, 1D CNNs have been proposed, achieving state-of-the-art results in several applications ranging from personalized biomedical data classification to anomaly detection and classification.

**Figure 3:**
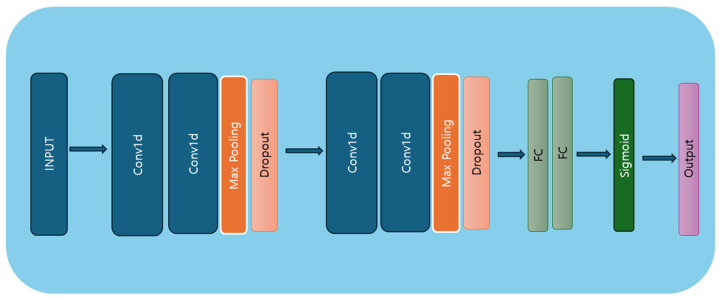
CNN model architecture diagram.

This study used 1D CNNs, with the model architecture presented in Figure 3.The architecture comprises four convolutional layers, two dropout layers, two max-pooling layers, and two dense layers (linear layers). The first CNN layer comprises 32 filters with a kernel size of 5, a stride of 1, and no padding, followed by an ReLU activation function [36]. The first layer accepts input data in the shape of batch size, number of features, and sequence length. The second convolutional layer has 64 filters, a kernel size of 3, a stride of 1, and a dropout layer [37] with a dropout rate of 0.5, which deactivates 50% of neurons during training. The third convolutional layer has 128 filters, a kernel size of 5, a stride of 1, and no padding, followed by ReLU activation. The fourth convolutional layer also has 128 filters, but uses a kernel size of 3, a stride of 1, and no padding. This layer is followed by a max-pooling layer with a kernel size of 2 and a stride of 2, and another dropout layer with a 0.5 dropout rate. Finally, two fully connected layers are applied, with the sigmoid activation function used in the last layer for predicting the target variable. The model architecture is outlined in Algorithm 1.
**Algorithm 1** Convolutional neural network modelX input shape: (batch_size, num_features, sequence length)
Model <- class CNNModel (nn.Module): # Initialization 1. Conv1_layer = nn.Sequential (nn.Conv1d) input size, output size = 32, kernel size = 5, stride = 1, padding = 0), nn.ReLU) 2. Conv2_layer = nn.Sequential nn.Conv1d (input size = 32, output size = 64, kernel size = 5, stride = 1, padding = 0), nn.ReLU, nn.MaxPool1d (kernel size = 3, stride = 1), dropout_layer = nn.Dropout (rate = 0.5) 3. Conv3_layer = nn.Sequential nn.Conv1d (input size = 64, output size = 128, kernel size = 5, stride = 1, padding = 0), nn.ReLU 4. Conv4_layer = nn.Sequential nn.Conv1d (input size = 128, output size = 128, kernel size = 3, stride = 1, padding = 0), nn.ReLU, nn.MaxPool1d (kernel size = 2, stride = 2), dropout_layer = nn.Dropout (rate = 0.5) 5. fully connected layer = nn.Linear (input size, output size) 6. fully connected layer2 = nn.Linear (input size, number of class)

#### 3.5.2. Long Short-Term Memory

**Figure 4:**
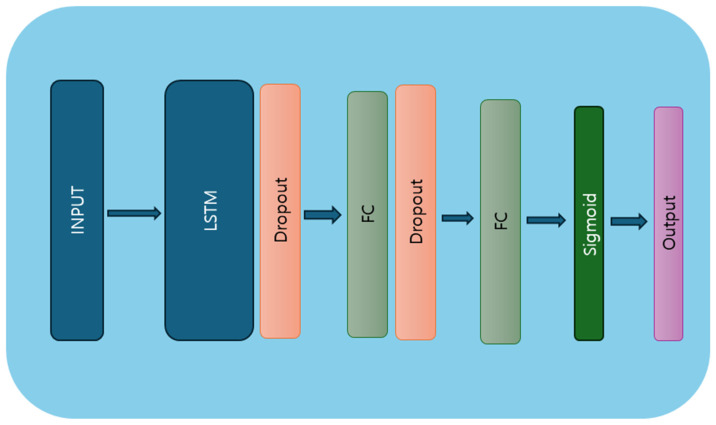
LSTM model architecture diagram.

Recurrent neural networks (RNNs) represent a class of artificial neural networks specifically designed to handle sequential data with temporal dependencies. Unlike traditional feedforward neural networks, RNNs possess internal memory mechanisms, enabling them to retain past input information and influence future predictions. This unique architecture makes RNNs well suited for a wide range of sequential data tasks, including natural language processing, time series prediction, and classification. However, RNNs or very deep networks are difficult to train, as they often suffer from issues like exploding or vanishing gradients. To overcome this challenge, when learning long-term dependencies, the LSTM architecture was proposed. The learning ability of LSTM impacted several fields from both a practical and theoretical perspective, leading it to become a state-of-the-art model. The LSTM model architecture is presented in Figure 4. It consists of an LSTM layer that takes batch size, sequence length, and number of channels as input data, with the number of channels representing the number of features in the data. This is followed by four layers, 128 hidden neurons, a dropout layer, and two dense layers. The input data are passed to the LSTM layer, followed by a dense layer. A dropout rate of 0.5 is then applied, turning off 50% of the neurons during the training stage to prevent the model from overfitting. The dropout layer is followed by a dense layer, where the sigmoid activation function is applied on the last output element for the prediction of the target variable. The model architecture is outlined in Algorithm 2.
**Algorithm 2** LSTM modelInput X input shape: (batch_size, sequence_length, num_features) # Define the LSTM model class Model <- class LSTMModel(nn.Module): 1. Create an LSTM layer with input size, hidden size, and number of layers. 2. Create a fully connected layer (fc) with output size, typically for regression tasks. 3. Implement a dropout layer to mitigate overfitting. 4. Apply an activation function, specifically sigmoid, to the final fully connected layer.

#### 3.5.3. Hybrid Model

**Figure 5:**
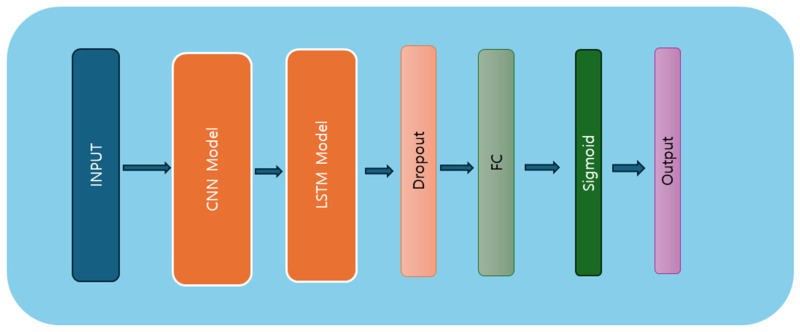
Hybrid model architecture diagram.

We inspired ourselves from the DeepConvLSTM [38] for implementing this hybrid model. The model constructed herein integrates both the DCNN and the LSTM layers, blending spatial and temporal feature extraction capabilities. The hybrid model architecture is presented in Figure 5. Comprising four convolutional layers and two LSTM layers, the model exhibits a multi-step process to ingest and analyze sequential data. Initially, the input data undergo convolutional operations across multiple layers, leveraging kernel-based feature extraction to discern spatial patterns. Subsequently, the processed features are fed into the LSTM layers, where temporal dependencies are captured and learned iteratively over sequential input sequences. The LSTM architecture, characterized by its memory cell and gate mechanisms, facilitates the retention and propagation of information across time steps, enabling the model to discern intricate temporal dynamics within the data. Following LSTM processing, the model employs fully connected layers to distill the learned representations into predictive outputs. Regularization techniques, including dropouts, are employed to mitigate overfitting and enhance model generalization. Ultimately, the model yields predictions via a final sigmoid activation function, encapsulating the culmination of feature extraction and temporal analysis. The model architecture is presented in Algorithm 3.
**Algorithm 3** CNN–LSTM Input X input shape: (batch_size, num_features, sequence length) # Define the Hybrid model class Model <- class HybridModel (nn.Module): # CNN architecture # Initialization 1. Conv1_layer = nn.Sequential (nn.Conv1d)      input size, output size = 128, kernel size = 5, stride = 1, padding = 0, nn.ReLU 2. Conv2_layer = nn.Sequential (nn.Conv1d)         input size = 128, output size = 128, kernel size = 5, stride = 1, padding = 0, nn.ReLU, nn.MaxPool1d (kernel size = 3) 3. dropout_layer = nn.Dropout (rate = 0.5) 4. Conv3_layer = nn.Sequential (nn.Conv1d)        input size, output size = 128, kernel size = 5, stride = 1, padding = 0, nn.ReLU 5. Conv2_layer = nn.Sequential (nn.Conv1d)        input size = 128, output size = 128, kernel size = 5, stride = 1, padding = 0, nn.ReLU, nn.MaxPool1d (kernel size = 3) 6. dropout_layer = nn.Dropout (rate = 0.5) 7. fully connected layer = nn.Linear (input size, output size) 8. fully connected layer2 = nn.Linear (input size, output size)  # LSTM architecture 1. Create an LSTM layer with input size, hidden size, and number of layers. 2. fully connected layer = nn.Linear (output size, output size)  # Concatenate 1 . Concatenate output size from CNN layers and LSTM layers. 2 . Apply the sigmoid activation function to the concatenated output. 3 . Return the final output.

#### 3.5.4. Training and Evaluation

The dataset used in this study is a sample (n = 173) of data collected from first-year high school students. Among the collected datasets, 4 (2%) belong to the underweight category, 111 (64%) to the normal category, 36 (20%) to the overweight category, and 22 (12%) to the obese category. In response to the heavy imbalance between the categories, we first combined the underweight and normal-weight groups and then merged the overweight and obese groups, resulting in two categories of data—the underweight/normal group (n = 115) representing 66% and the overweight/obese group (n = 58) representing 34% of the data—creating a binary classification problem (normal vs. obese). Overall, 80% of the data was used for training and the remaining 20% for testing the performance of the models.

In addition, for training the models, identification values were assigned for each group, “0” for the normal group and “1” for the overweight/obese group. The models and the training step were implemented using the PyTorch framework [39]. Each model was compiled using the binary cross-entropy loss [40] function, which is well-suited for binary classification tasks, and the Adam [41] (adaptive moment estimation) optimizer, used to minimize the cost of the model, was chosen for its efficiency in handling sparse gradients and its adaptive learning rate capabilities. The learning rate was set to 0.003 and the training process was conducted over 50 epochs.

## 4. Results

When the data were trained using the LSTM model, a rapid decrease in loss was observed within the first five epochs, along with high accuracy on both the training and the validation set. This highlights that the model was able to identify meaningful patterns in both the normal group and the overweight/obese group. Similarly, when the data were trained on the CNN and the hybrid model, a rapid decrease in loss and a rapid increase in accuracy, was observed indicating that both models effectively extracted meaningful patterns from the data across both groups.

**Figure 6:**
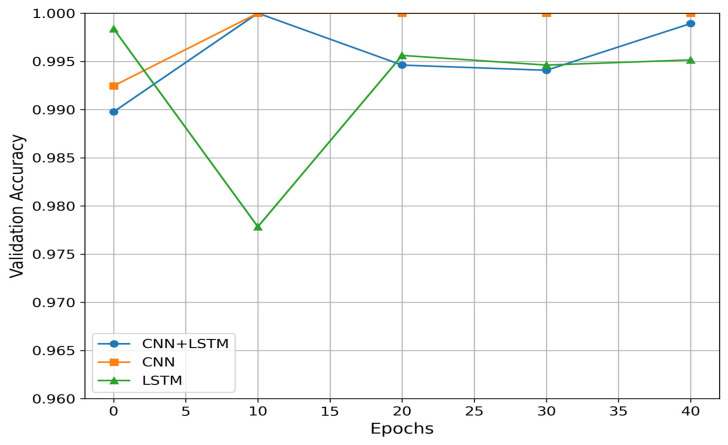
Validation accuracy per model.

During the training phase, the best model with the lowest loss on the validation set was saved and used to perform predictions for unseen test data. For the CNN model, the best validation loss was obtained after 26 iterations, with a value of 2.98 × 10^−7^ and an accuracy of 99.99%. For the LSTM model, the best validation loss was achieved after 38 iterations, with a value of 2.32 × 10^−5^ and an accuracy of 99.95%. Lastly the hybrid model reached its best validation loss after 10 iterations, with a value of 2.98 × 10^−7^ and an accuracy of 99.99%. The results of the validation process are presented in Table 2. Figure 6. shows the validation accuracy of each model.

The predictions for the test data are presented in Table 3. The testing data consisted 35 datasets, with 23 subjects in the normal group and 12 subjects in the overweight/obesity group. As in the training set, we applied data windowing extracting 100 samples with 50% overlap. The predictions for the test data using the deep convolutional neural network model yielded an accuracy of 95.81%, the LSTM model achieved an accuracy of 96.31%, and the hybrid model reached an accuracy of 97%. The hybrid model consistently outperformed the CNN and LSTM models, likely due to its ability to capture both spatial and temporal features in the gait data. The CNN layers extract spatial features from the input sequences, while the LSTM layers capture temporal dependencies, leading to superior overall performance. Although the CNN model achieved strong results during training, it struggled to generalize as well on unseen data, likely because it lacked the ability to fully account for the sequential nature of the gait data, which explains its slightly lower performance on the test data. The LSTM model performed well in capturing temporal dependencies, but without the spatial feature extraction capabilities of the CNN, its performance was marginally lower than the hybrid model. This suggests that the combination of spatial and temporal feature extraction is crucial for better accuracy in identifying overweight/obesity from gait data.

## 5. Discussion

This study explored the use of three deep learning models for addressing obesity recognition in adolescents using gait data collected through smartphone inertial sensors. The three models, namely, CNN, LSTM, and a hybrid model, were trained on data from 138 participants, with 92 in the normal group and 46 in the overweight and obese group. They were tested on 35 participants’ data, with 23 in the normal group and 12 in the overweight and obese groups. The results indicated that the hybrid model outperformed both CNN and LSTM models in classifying obesity based on gait patterns. This suggests that integrating both spatial and temporal feature extraction is critical for capturing the complex differences between normal and overweight/obese gait. The hybrid model’s superior performance may be due to its ability to process the sequential nature of gait data while also recognizing spatial features within each segment. Notably, while the CNN model achieved great results on the training set, it did not generalize as well on the test set as the LSTM model, possibly due to its limitation in handling temporal dependencies inherent in sequential data. Our findings are consistent with those of Steinberg et al. [28], who also found significant differences in gait patterns between overweight/obese and normal-weight individuals. However, while Steinberg’s study focused primarily on hip joint angles and knee trajectories, our study extends these findings by demonstrating that these differences can be effectively captured using smartphone-based inertial sensors combined with deep learning models. In contrast, Murtha et al. [27] observed limited success in predicting weight gain using health records, suggesting that static health data may not capture the dynamic characteristics needed for accurate obesity prediction. Building on the work of Lee et al. [31], our study shows that the CNN model alone is insufficient for capturing all the gait patterns and characteristics between normal and overweight/obese groups. However, by using LSTM and a hybrid model, we achieved better accuracy. Despite these promising results, this study has several limitations that should be considered. First the sample, particularly in the obese category, was relatively small, which may limit the generalizability of our findings. Future work should aim to include larger and more diverse samples to validate these results. Additionally, the use of a single smartphone model for data collection may introduce device-specific biases, so testing across multiple devices would be beneficial to ensure robustness. Another limitation is the exclusion of participants with potentially abnormal gait conditions. While this was necessary to focus on obesity-related gait differences, it also means that our findings may not apply to populations with other gait-affecting conditions.

## 6. Conclusion

This study explored the relationship between BMI and walking patterns. Building on the work of Lee et al. [31], walking data were collected from 244 first-year high school students using a mobile health application, with a sample of 173 students included in this study. The smartphone was placed inside a pocket bag and worn at the center of the body by the participants. They were instructed to walk over a 78-foot course in a straight line and then return to the starting point. We used the smartphone’s inertial sensor data, specifically the nine-channel rotation matrix from the rotation vector, to analyze the gait patterns. Three deep learning algorithms were employed: convolutional neural network (CNN), long short-term memory (LSTM) network, and a hybrid model combining CNN and LSTM. The highest accuracy was achieved with the hybrid model, at 97%, followed by the LSTM model at 96.31% and the CNN model at 95.81%. All of our models outperformed the results obtained by Lee et al. [31].

## Figures and Tables

**Table 1:** Demographic information of the student participants.

Characteristics	Overall (*n* = 173)	BMI 1 (≤5%)	BMI2 (>5% and <85%)	BMI3 (>85% and <95%)	BMI4 (>95%)
**Gender**					
Female	86	2	58	22	8
Male	84	2	53	14	14
**Race**					
Hispanic	18	0	18	4	1
White	136	4	86	27	20
Black	3	0	0	3	0
Asian	4	0	2	1	1
Other	12	0	5	1	0
Total	173	4	111	36	22

**Table 2:** Results of evaluation metrics on validation set.

Model	Loss	Accuracy
CNN	2.98 × 10^−7^	0.9999
LSTM	2.32 × 10^−5^	0.9995
CNN–LSTM	2.98e × 10^−7^	0.9999

**Table 3:** Results of evaluation metrics on test set.

Model	Accuracy	Precision	Recall	F1
Lee et al. [31]	90.5	X	X	X
CNN (ours)	**95.81**	0.99	87.78	93.49
LSTM (ours)	**96.31**	0.99	89.23	94.31
CNN–LSTM (ours)	**97**	0.99	91.23	95.41

Bold values highlight superior results compared to previous studies.

## Data Availability

No new data were created or analyzed in this study. Data sharing is not applicable to this article.
